# Concomitant medications associated with ischemic, hypertensive, and arrhythmic events in MDMA users in FDA adverse event reporting system

**DOI:** 10.3389/fpsyt.2023.1149766

**Published:** 2023-05-18

**Authors:** Tigran Makunts, Diane Dahill, Lisa Jerome, Alberdina de Boer, Ruben Abagyan

**Affiliations:** ^1^MAPS Public Benefit Corporation, San Jose, CA, United States; ^2^Skaggs School of Pharmacy and Pharmaceutical Sciences, University of California, San Diego, San Diego, CA, United States; ^3^Tulip Medical Consulting LLC, Port Townsend, WA, United States

**Keywords:** 3,4-methylenedioxymethamphetamine, MDMA, adverse events, cardiovascular, schaemia, hypertension, arrhythmia

## Abstract

3,4-Methylenedioxymethamphetamine (MDMA) is currently being investigated as an adjunctive treatment to therapy for posttraumatic stress and other anxiety related disorders in clinical trials. Within the next few years MDMA-assisted therapy is projected for approval by regulatory authorities. MDMA’s primary mechanism of action includes modulation of monoamine signaling by increasing release and inhibiting reuptake of serotonin, norepinephrine, and, to a lesser extent, dopamine. This pharmacology affects sympathomimetic physiology. In controlled trials, special attention has been given to cardiovascular adverse events (AEs), because transient increases in heart rate and blood pressure have been observed during the MDMA-assisted therapy sessions. Finding and quantifying the potential drivers of cardiac AEs in clinical trials is difficult since only a relatively small number of participants have been included in these studies, and a limited set of allowed concomitant drugs has been studied. In this study a more diverse set of reports from the FDA Adverse Event Reporting System was surveyed. We found 17 cases of cardiovascular AEs, in which the individuals had taken one or more substances in addition to MDMA. Interestingly, all of those concomitant medications and illicit substances, including opioids, stimulants, anticholinergics, and amphetamines, had been previously associated with cardiovascular AEs. Furthermore, in none of the reports MDMA was marked as the primary suspect.

## Introduction

3,4-methylenedioxymethamphetamine also known as MDMA or its street name Ecstasy, is currently a schedule I controlled substance in the United States and the European Union, and a Class A substance in the United Kingdom. There is growing interest in MDMA’s utilization in psychiatry based on promising efficacy and safety findings from multiple controlled clinical trials including a Phase 3 study for MDMA assisted therapy for post-traumatic stress disorder (PTSD) (1–5).

MDMA’s psychoactive effects are due to its complex pharmacology, including modulation of release and reuptake of serotonin, norepinephrine, and dopamine (6–9), and an increase in oxytocin levels (10). The efficacy of MDMA in PTSD treatment is attributed to supporting fear-extinction learning an increased ability to confront adverse memories, and improved social and interpersonal interactions (5, 11–13).

However, due to the monoamine neurotransmission modulation, cardiovascular physiology may be affected as well (14–16). Arrhythmia-related adverse events (AEs), in addition to hypertensive and ischemia-related AEs have been reported in literature (17–20). In controlled clinical trials in both healthy volunteers and patients with PTSD, AEs of transient increases in blood pressure and heart rate were observed along with muscle tightness, decreased appetite, nausea, hyperhidrosis, and feeling cold (4, 21).

Considering the polypharmacy often present in PTSD populations due to other comorbid conditions such as substance use (22, 23), anxiety (24), depression (25), sleep (26) and pain disorders (27), and the respective treatment drugs, further MDMA AE evaluation is warranted using the FAERS database. This is particularly important since the clinical trials excluded many of these medications, and there is potential for drug–drug interactions (28).

In this study, we evaluated arrhythmic, ischemic, and hypertensive AE reports in MDMA users from the FDA Adverse Event Reporting System (FAERS). These AEs were reviewed in submissions where MDMA was reported to be used alone or with additional therapeutics or illicit substances. The contribution of concomitant drugs and substances to the risk of cardiovascular AEs was evaluated.

## Methods

### FDA adverse event reporting system

FAERS is a repository of AEs submitted to the FDA through MedWatch (Forms 3,500/3500A) (29) by consumers, legal representatives, healthcare professionals, sponsors and manufacturers.

FAERS was initially intended for post-marketing drug and biologic safety surveillance to detect and re-evaluate drug safety signals that may have been missed in smaller scale controlled trials. However, the database includes reports of drugs still under investigation and Schedule I substances, making it a useful resource to evaluate safety of substances not yet approved by the FDA and other regulatory authorities. Reporting use of unapproved or illegal substances is important, since those agents may be the culprits of the adverse events wrongly attributed to concomitant therapeutics.

### Combining and normalizing data sets

FAERS/AERS quarterly data sets, each including a data subset (demographics, drug, indication, outcome, reaction, report source), were downloaded individually from the FDA public repository in dollar sign-separated text format (30–32). At the time of the study FAERS/AERS contained 18,274,795 reports from January 2004 to September 2022. It was convenient to standardize the multiple data tables into a unified single table structure. A set of Unix shell scripts was used for data restructuring and filtering (33). The partially missing fields, relevant to the current analysis, in the MDMA reports were only in the demographic section (age, weight, sex), and were comparable to the rest of the database.

### Case selection

**Figure 1:**
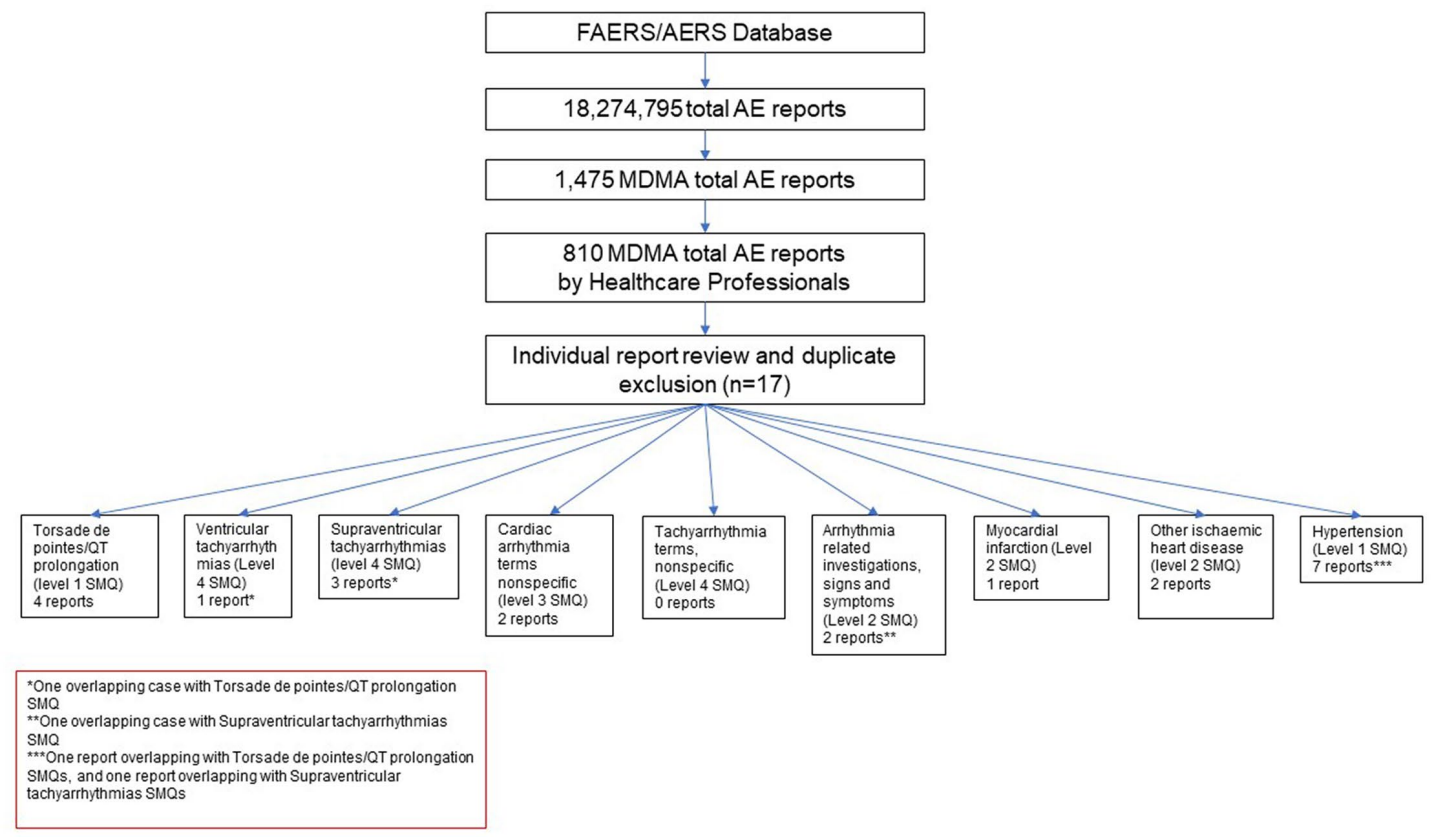
MDMA Cardiac AE report selection flowchart.

Cases where one of the reported drugs included the terms methylenedioxymethamphetamine, 3,4-methylenedioxymethamphetamine, midomaphetamine, midomafetamine, MDMA, ecstasy, were selected into the MDMA cohort for review. A total of 1,475 reports were selected. Further, cases where reports were submitted by a healthcare professional were selected to avoid reporting bias and add clinical relevance to the reports. The resulting cohort was queried for AEs with Preferred Terms (PT) based on the following Standardized MedDRA queries (SMQs) (34) consisting of a series of specific terms intended to select key symptoms or diagnoses: Torsade de pointes/QT prolongation (level 1 SMQ), Arrhythmia related investigations, signs and symptoms (Level 2 SMQ), Cardiac arrhythmia terms nonspecific (level 3 SMQ), Supraventricular tachyarrhythmias (level 4 SMQ), Hypertension (Level 1 SMQ), Ventricular tachyarrhythmias (Level 4 SMQ), Tachyarrhythmia terms, nonspecific (Level 4 SMQ). A comprehensive list of both narrow and broad scope SMQ PTs used in the query can be found in Supplementary Table S1. Cases were selected if at least one narrow scope PT or two or more broad scope PTs were reported. Reports were further individually reviewed to exclude duplicate submissions from multiple reporting sources resulting in 17 unique reports with AEs of interest (Figure 1). No other inclusion and exclusion parameters were applied in the case selection. All of the screened cases were included in the study (*n* = 17).

## Results

A total of 17 unique cases were reviewed in this study. There were no reports where MDMA was taken as a single agent and ischemic, hypertensive, or arrhythmic AEs were reported. All cases included concomitant medications with known associated cardiac function abnormalities. There were a total of four cases matching the *Torsade de pointes/QT prolongation* SMQ search criteria (Supplementary Table S1). In *all* of the cases, MDMA was taken with concomitant medications (SSRIs, antihistamines/anticholinergics, amphetamines) with known effects on cardiac function (Table 1). These cases included one report of ventricular fibrillation which matched the *Ventricular tachyarrhythmias* SMQ search query (Table 2). The *Supraventricular tachyarrhythmia* SMQ query produced three reports with one report overlapping with *Torsade de pointes/QT prolongation* SMQ terms (Table 3). The *Cardiac arrhythmia terms nonspecific* SMQ search produced two reports with concomitant reported cocaine, opioid, benzodiazepine, gamma hydroxybutyrate, and cannabis use (Table 4). There were two reports in the *Arrhythmia related investigations, signs and symptoms* query, both based on three broad scope AE PTs (Supplementary Table S1), one of which (case #6) overlapped with the *Supraventricular tachyarrhythmia* query (Table 5). The *Myocardial infarction* SMQ based search produced one report of an AE of troponin increased, where MDMA was taken with clozapine (Table 6). The *Tachyarrhythmia terms nonspecific* SMQ search produced no reports. The *Other ischaemic heart disease* query found two unique reports and the ‘Hypertension’ query produced seven reports with two overlaps with *Torsade de pointes/QT prolongation* and *Supraventricular tachyarrhythmias SMQs* (Table 7). There were seven *Hypertension* SMQ cases with two reports overlapping with *Torsade de pointes/QT prolongation* and *Supraventricular tachyarrhythmias* SMQs (Table 8).

**Table 1:** Torsade de pointes/QT prolongation (level 1 SMQ) cases in FAERS/AERS.

Case # (number of duplicates)	Age	Sex	Country	Concomitant medications	Adverse events	Outcome	Reported by
1 (6)	15	F	DE	ps:cetirizine ss:ecstasy	Drug interaction **Electrocardiogram qt prolonged** Toxicity to various agents Urine amphetamine positive	HO	MD HP
2 (2)	25	M	AU	ps:sertraline ss:midomafetamine	Abdominal pain upper Aggression Alanine aminotransferase increased Aspartate aminotransferase increased Blood potassium decreased **Blood pressure increased** Decreased appetite Disorientation Drug abuse **Electrocardiogram qt prolonged** **Heart rate increased** Muscle rigidity Nausea Oxygen saturation decreased Serotonin syndrome **Sinus tachycardia** Vomiting Weight decreased	OT	HP
3	25	M	US	ps:sertraline ss:cocaine ss:midomafetamine c:st john’s wort	Abdominal pain upper Aggression Alanine aminotransferase increased Aspartate aminotransferase increased Blood potassium decreased Decreased appetite Disorientation Drug abuse **Electrocardiogram qt prolonged** Nausea Oxygen saturation decreased Serotonin syndrome Vomiting Weight decreased	OT	HP
4	unk	unk	TR	ps:amphetamine ss:mdma	Cardiac arrest Cardioversion Coma scale abnormal Hyperthermia Hypotension Seizure Toxicity to various agents **Ventricular fibrillation**	HO OT	MD

unk, unknown; M, male; F, female; DE, Germany; AU, Australia; US, United States; TR, turkey; ps, primary suspect; ss, secondary suspect; c, concomitant; HO, hospitalization; OT, other serious (important medical event); HP, health professional; MD, physician.The bold values are the terms of the adverse events related to the study focus (hypertensive, ischaemic, and arrhythmic).

**Table 2:** Ventricular tachyarrhythmias (Level 4 SMQ) cases in FAERS/AERS.

Case #	Age	Sex	Country	Concomitant medications	Adverse events	Outcome	Reported by
4*	unk	unk	TR	ps:amphetamine ss:mdma	Cardiac arrest Cardioversion Coma scale abnormal Hyperthermia Hypotension Seizure Toxicity to various agents **Ventricular Fibrillation**	HO OT	MD

* This case also matches the Torsade de pointes/QT prolongation SMQ criteria (see Table 1).

unk, unknown; TR, turkey; ps, primary suspect; ss, secondary suspect; HO, hospitalization; OT, other serious (important medical event); MD, physician.The bold values are the terms of the adverse events related to the study focus (hypertensive, ischaemic, and arrhythmic).

**Table 3:** Supraventricular tachyarrhythmias (level 4 SMQ) cases in FAERS/AERS.

Case #(number of duplicates)	Age	Sex	Country	Concomitant medications	Adverse events	Outcome	Reported by
5(2)	19	F	FR	ps:fluoxetine ss:cannabis ss:citalopram c:3 4 methylenedioxymethamphetamine c:cocaine c:methylphenidate	Agitation **Blood pressure increased** Clonus Coma scale abnormal Hallucination visual Hypertonia Respiratory acidosis **Sinus tachycardia** Tonic convulsion Toxicity to various agents	HO OT	MD
6(3)	55	F	IT	ps:acetaminophen ss:bromazepam ss:carbamazepine ss:trazodone c:methylenedioxymethamphetamine c:morphine c: tramadol c: codeine c: naloxone c: pregabalin	**Atrial fibrillation** Bradycardia Bradypnoea Hepatitis acute Hypokalaemia Hypotension Hypothermia Loss of consciousness Mydriasis Overdose Product use in unapproved indication	HO LT OT	HP
2(2)*	25	M	AU	ps:sertraline: ss:midomafetamine	Abdominal pain upper Aggression Alanine aminotransferase increased Aspartate aminotransferase increased Blood potassium decreased **Blood pressure increased** Decreased appetite Disorientation Drug abuse **Electrocardiogram qt prolonged** **Heart rate increased** Muscle rigidity Nausea Oxygen saturation decreased Serotonin syndrome **Sinus tachycardia** Vomiting Weight decreased	OT	HP

* This case also matches the Torsade de pointes/QT prolongation SMQ criteria (see Table 1).

M, male; F, female; FR, France; IT, Italy; AU, Australia; ps, primary suspect; ss, secondary suspect; c, concomitant; HO, hospitalization; LT, life threatening; OT, other serious (important medical event); HP, health professional; MD, physician.The bold values are the terms of the adverse events related to the study focus (hypertensive, ischaemic, and arrhythmic).

**Table 4:** Cardiac arrhythmia terms nonspecific (level 3 SMQ) cases in FAERS/AERS.

Case #	Age	Sex	Country	Concomitant medications	Adverse events	Outcome	Reported by
7	unk	unk	GB	ps:fentanyl ss:alprazolam ss:clonazepam ss:cocaine ss:diazepam ss:gamma hydroxybutyrate ss:heroin ss:methylenedioxymethamphetamine ss:oxazepam ss:tramadol HCl	**Arrhythmia** Cardiac arrest Coma scale abnormal Death Drug abuse Seizure Toxicity to various agents	DE HO OT	HP
8	23	M	FR	ps:pregabalin ss:cannabis sativa subsp. indica top ss:cocaine ss:midomafetamine	Coma Drug abuse **Heart rate irregular** Partial seizures	HO	MD

unk, unknown; M, male; F, female; FR, France; IT, Italy; AU, Australia; ps, primary suspect; ss, secondary suspect; c, concomitant; HO, hospitalization; LT, life threatening; OT, other serious (important medical event); HP, health professional; MD, physician.The bold values are the terms of the adverse events related to the study focus (hypertensive, ischaemic, and arrhythmic).

**Table 5:** Arrhythmia related investigations, signs, and symptoms (Level 2 SMQ) cases in FAERS/AERS.

Case #(number of duplicates)	Age	Sex	Country	Concomitant medications	Adverse events	Outcome	Reported by
6 (3)*	55	F	IT	ps:acetaminophen ss:bromazepam ss:carbamazepine ss:trazodone c:methylenedioxymethamphetamine c:morphine	Atrial fibrillation **Bradycardia** Bradypnoea Hepatitis acute Hypokalaemia Hypotension Hypothermia **Loss of consciousness** Mydriasis Overdose Product use in unapproved indication	HO LT OT	HP
9	unk	unk	GB	ps:acetaminophenss:amphetamine ss:aspirin ss:buprenorphine ss:cannabis ss:cocaine ss:codeine ss:diamorphine ss:diclofenac ss:ecstasy ss:ibuprofen ss:methadone ss:morphine	Abdominal pain Abdominal symptom Accident at work Adverse event Aggression Back pain Cardiac disorder **Cardio respiratory arrest** Cerebrovascular accident Chest pain Depressed level of consciousness Diabetic complication Exposure to unspecified agent Fall Gunshot wound Hemorrhage Headache Injury Laceration **Loss of consciousness** Malaise Multiple allergies Overdose Physical assault psychiatric symptom Respiratory disorder Respiratory distress Road traffic accident Seizure Sexual abuse Stab wound Substance abuse Suicidal ideation **Syncope** Toxicity to various agents	HO OT	MD

* This case also matches the Supraventricular tachyarrhythmias SMQ criteria (see Table 3).

unk, unknown; F, female; GB, Great Britain; IT, Italy; ps, primary suspect; ss, secondary suspect; c, concomitant; DE, death; HO, hospitalization; OT, other serious (important medical event); LT, life threatening; HP, health professional; MD, physician.The bold values are the terms of the adverse events related to the study focus (hypertensive, ischaemic, and arrhythmic).

**Table 6:** Myocardial infarction (Level 2 SMQ) cases in FAERS/AERS.

Case #	Age	Sex	Country	Concomitant medications	Adverse events	Outcome	Reported by
10	unk	M	AU	ps:clozapine ss:ecstasy	**Troponin increased**	HO	MD

unk, unknown; M, male; AU, Australia; ps, primary suspect; ss, secondary suspect; c, concomitant; HO, hospitalization; MD, physician.The bold values are the terms of the adverse events related to the study focus (hypertensive, ischaemic, and arrhythmic).

**Table 7:** Other ischaemic heart disease (level 2 SMQ).

Case # (number of duplicates)	Age	Sex	Country	Concomitant medications	Adverse events	Outcome	Reported by
11(3)	unk	unk	AU	ps:fentanyl ss:alcohol ss:buprenorphine ss:cannabinol ss:cocaine ss:codeine ss:hydromorphone ss:methadone ss:methamphetamine ss:midomafetamine ss:morphine ss:olanzapine ss:oxycodone ss:promethazine ss:quetiapine ss:tapentadol ss:tramadol HCl	**Arteriosclerosis coronary Artery** Aspiration Asthma Cardiac valve disease Cardiomegaly Cardiomyopathy Emphysema Fibrosis Hepatic cirrhosis Hepatic fibrosis Hepatic hypertrophy Hepatic steatosis Hepatitis Intentional self injury Kidney fibrosis Nephrosclerosis Overdose Pneumonia Pulmonary oedema Toxicity to various agents Ventricular hypertrophy Death	DE OT	HP
12(6)	22	F	AU	ps:morphine sulfate ss:acetaminophen ss:furosemide ss:methadone HCl ss:methamphetamine ss:methylenedioxymethamphetamine ss:metoclopramide	Hepatitis c **Myocardial ischaemia** Pulmonary oedema Toxicity to various agents Death	DE OT	MD

unk, unknown; M, male; AU, Australia; ps, primary suspect; ss, secondary suspect; c, concomitant; HO, hospitalization; MD, physician.The bold values are the terms of the adverse events related to the study focus (hypertensive, ischaemic, and arrhythmic).

**Table 8:** Hypertension (Level 1 SMQ) cases in FAERS/AERS.

Case # (number of duplicates)	Age	Sex	Country	Concomitant medications	Adverse events	Outcome	Reported by
2 (2)*	25	M	AU	ps:sertraline ss:midomafetaminea	Abdominal pain upper Aggression Alanine aminotransferase Increased Aspartate aminotransferase increased Blood potassium decreased **Blood pressure increased** Decreased appetite Disorientation Drug abuse Electrocardiogram qt prolonged Heart rate increased Muscle rigidity Nausea Oxygen saturation decreased Serotonin syndrome Sinus tachycardia Vomiting Weight decreased	OT	HP
13	44	M	FR	ps:diazepam ss:alcohol ss:cannabis ss:cocaine ss:lsd ss:mdma ss:tabacum inhalation	Drug abuse Gamma glutamyltransferase increased **Hypertension** Hypertriglyceridaemia	HO	MD
14	24	F	FR	ps:alprazolam ss:amphetamine ss:midomafetamine	**Hypertension** Somnolence Suicide attempt Tachycardia	OT	PH
15 (3)	32	M	FR	ps:pregabalin ss:midomafetamine ss:clonazepam ss:prazepam	Drug abuse **Hypertension** Somnolence Victim of crime	OT	PH
16	unk	unk	unk	ps:sodium valproate ss:benzocaine ss:mdai ss:methylenedioxymethamphetamine ss:mirtazapine c:amoxicillin c:levomepromazine c:orphenadrine	**Blood pressure increased** Cyanosis central Dizziness Malaise Methaemoglobinaemia Off label use Oxygen saturation decreased PO2 increased Respiratory rate increased	HO	MD
5(2)**	19	F	FR	ps:fluoxetine ss:cannabis ss:citalopram c:3 4 methylenedioxymethamphetamine c:cocaine c:methylphenidate	Agitation **Blood pressure increased** Clonus Coma scale abnormal Hallucination visual Hypertonia Respiratory acidosis **Sinus tachycardia** Tonic convulsion Toxicity to various agents	HO OT	MD
17	unk	M	FR	ps:buprenorphine ss:alcohol ss:methylenedioxymethamphetamine	Agitation Delirium drug abuse **Hypertension** Injection site inflammation|	HO	PH

* This case also matches the Torsade de pointes/QT prolongation SMQ criteria (see Table 1).

** This case also matches the Supraventricular tachyarrhythmias SMQs criteria (See Table 3).

unk, unknown; M, male; F, female; FR, France; AU, Australie; ps, primary suspect; ss, secondary suspect; c, concomitant; HO, hospitalization; OT, other serious (important medical event); HP, health professional; PH, pharmacist; MD-physician.The bold values are the terms of the adverse events related to the study focus (hypertensive, ischaemic, and arrhythmic).

The summary of all of the cases, with the concomitant medications organized by class, is provided in Table 9.

**Table 9:** Concomitant drugs associated with cardiac function related AEs in MDMA cases summarized by class.

Report number	1	2	3	4	5	6	7	8	9	10	11	12	13	14	15	16	17
MDMA	*	*	*	*	*	*	*	*	*	*	*	*	*	*	*	*	*
Opioid agonists/antagonists						****	***		*****		***** ****	**					*
Amphetamines, NET/DAT inhibitors				*	*						*	*		*			
Benzodiazepines						*	***						*	*			
Cocaine			*		*		*	*	*		*		*		*		
Hydroxybutyrate							*										
Antihistamines/ anticholinergics	*										*					**	
SSRIs/SSRAs/ antidepressants		*	**		**	*										**	
Gabapentinoids						*		*							*		
Alcohol											*		*				*
Cannabinoids					*			*	*		*		*				
Antipsychotics										*	**						
LSD													*				
NSAIDS									***								

The number of* corresponds to the number of drugs in the listed drug class included in the report. NET, norepinephrine transporter; DAT, dopamine transporter; SSRI, selective serotonin reuptake inhibitor; SSRA, selective serotonin releasing agent; LSD, lysergic acid diethylamide; NSAID, nonsteroidal anti-inflammatory drug.The bold values are the terms of the adverse events related to the study focus (hypertensive, ischaemic, and arrhythmic).

## Discussion

In this study, we evaluated AEs related to arrhythmias, hypertension, and ischemia in MDMA reports from the FDA adverse Event Reporting System (See Supplementary Table S1). There were no reports of those AEs in cases where MDMA was the sole reported drug. A limited number of 17 cases associated with MDMA use, reported in the last ~18 years, were evaluated. Interestingly, in every single case, MDMA was not reported as the primary suspect of those AEs. Furthermore, in all the cases, all listed concomitant drugs except one, acetaminophen, had known cardiac function related effects and were marked as primary suspects. There were two unique acetaminophen overdose cases that reported MDMA as a concomitant drug. Additionally, 76% of the reports included two or more drugs or illicit substances associated with arrhythmic, ischemic, or hypertensive AEs. It is interesting to note that in majority of the cases, illicit substances were taken in combination with psychoactive prescription medications supporting previous observations that substance use and abuse are often comorbid with psychiatric disorders. While individuals with mental health are more susceptible to misuse/abuse of illicit substances, this correlation is more complex and multidimensional as misuse/abuse may themselves lead to psychiatric disorders (35, 36).

There is still the possibility that MDMA contributed to the cardiovascular AE due to its sympathomimetic mechanism of action or CYP2D6-mediated drug–drug interaction(s) (37, 38). However, it was neither a sole culprit nor a primary suspect in any of the reported cases. The United Nations Office of Drugs and Crime estimated the number of people who report MDMA/ecstasy use to be nearly 20 million people (39). Surprisingly, considering this number, the number of reported MDMA cases with cardiovascular AE in FAERS/AERS was surprisingly low. Despite the fact that MDMA, as a known sympathomimetic, transiently increases blood pressure which is also observed in clinical trials, the number of FAERS reports on hypertension is relatively low, supporting the transient nature of this observation.

*Study limitations.* Due to the voluntary nature of FAERS/AERS reporting, with the exception of spontaneous reports from sponsors/manufacturers, the data presented only represents a subset of actual cases and should not be confused with actual population frequencies. Additionally, since the manufacturing and distribution of MDMA is not regulated, it is not clear whether the reported compound was pure MDMA or if it was laced with another compound not caught by the conventional drug tests. Information on the ingested MDMA dose and how the presence of MDMA was evaluated is missing from the reports, since case narratives are kept confidential by the FDA due to privacy concerns. Detailed medical and psychiatric history of the individuals in the reports were also not available. However, all of the 17 cases presented in the study were submitted by healthcare professionals (*Form*-3,500), thus some level of clinical adjudication prior to reporting is expected.

## Data availability statement

The datasets presented in this study can be found in online repositories. The names of the repository/repositories and accession number(s) can be found below: https://fis.fda.gov/extensions/FPD-QDE-FAERS/FPD-QDE-FAERS.html.

## Ethics statement

Ethical approval was not provided for this study on human participants because Ethical review and approval was not required for the study on human participants in accordance with the local legislation and institutional requirements. Written informed consent from the participants’ legal guardian/next of kin was not required to participate in this study in accordance with the national legislation and the institutional requirements. Study utilized de-identified postmarketing data available online to the public. Written informed consent for participation was not required for this study in accordance with the national legislation and the institutional requirements.

## Author contributions

TM performed the research. TM, DD, LJ, AB, and RA designed the study, drafted the manuscript, and reviewed the final version. RA processed the data sets. All authors contributed to the article and approved the submitted version.

## Funding

The study was funded by MAPS Public Benefit Corporation and in part by Skaggs School of Pharmacy and Pharmaceutical Sciences, UC San Diego Health.

## Conflict of interest

TM, DD, and LJ were employed by MAPS Public Benefit Corporation. AB was employed by Tulip Medical Consulting LLC.

The remaining author declare that the research was conducted in the absence of any commercial or financial relationships that could be construed as a potential conflict of interest.

## Publisher’s note

All claims expressed in this article are solely those of the authors and do not necessarily represent those of their affiliated organizations, or those of the publisher, the editors and the reviewers. Any product that may be evaluated in this article, or claim that may be made by its manufacturer, is not guaranteed or endorsed by the publisher.
