# Supplementary material for: Concomitant medications associated with ischemic, hypertensive, and arrhythmic events in MDMA users in FDA adverse event reporting system
Source: Front Psychiatry. 2023 May 18;14:1149766. doi: 10.3389/fpsyt.2023.1149766 (PMC10233020; doi:10.3389/fpsyt.2023.1149766)
Supplement: Supplementary file 1 [file Table_1.DOCX]

**Concomitant medications associated with ischemic, hypertensive, and arrhythmic events in MDMA users in FDA Adverse Event Reporting System**

Tigran Makunts^1,2^*, Diane Dahill^1^, Lisa Jerome^1^, Alberdina de Boer^3^, Ruben Abagyan^2^

^1^MAPS Public Benefit Corporation, San Jose, CA, United States

^2^Skaggs School of Pharmacy and Pharmaceutical Sciences, University of California, San Diego, San Diego, CA, United States

^3^Tulip Medical Consulting LLC, Port Townsend, WA, United States

Correspondence to TM: tigran.makunts@mapsbcorp.com

**Supplement Table 1. Cardiovascular area AE PTs organized by SMQs.**

| **AESI area** | **SMQ** | **Narrow Scope PTs** | **Broad Scope PTs** |
| --- | --- | --- | --- |
| Cardiovascular (arrhythmic, ischaemic, hypertensive AEs) | Torsade de pointes/QT prolongation (level 1 SMQ) | Electrocardiogram QT interval abnormal  Electrocardiogram QT prolonged  Long QT syndrome  Torsade de pointes  Ventricular tachycardia | Arrhythmic storm  Cardiac arrest  Cardiac death  Cardiac fibrillation  Cardio-respiratory arrest  Electrocardiogram repolarization abnormality  Electrocardiogram U wave inversion  Electrocardiogram U wave present  Electrocardiogram U-wave abnormality  Loss of consciousness  Sudden cardiac death  Sudden death  Syncope  Ventricular arrhythmia  Ventricular fibrillation  Ventricular flutter  Ventricular tachyarrhythmia  Ventricular tachycardia |
|  | Arrhythmia related investigations, signs and symptoms (Level 2 SMQ) | Chronic incompetence  Early repolarization syndrome  Electrocardiogram repolarization abnormality  Electrocardiogram RR interval prolonged  Electrocardiogram U wave inversion  Electrocardiogram U wave present  Electrocardiogram U wave abnormality  Sudden cardiac death | Bezold-Jarisch reflex  Bradycardia  Cardiac arrest  Cardiac death  Cardiac telemetry abnormal  Cardio-respiratory arrest  Central bradycardia  Cerebrocardiac syndrome  Electrocardiogram abnormal  Electrocardiogram ambulatory abnormal  Electrocardiogram change  Heart rate abnormal  Heart rate decreased  Heart rate increased  Loss of consciousness  Palpitations  Rebound tachycardia  Sudden death  Syncope  Tachycardia  Tachycardia paroxysmal |
|  | Cardiac arrhythmia terms nonspecific (level 3 SMQ) | Arrhythmia  Heart alternation  Heart rate irregular  Holiday heart syndrome  Paroxysmal arrhythmia  Pulseless electrical activity  Withdrawal arrhythmia  Reperfusion arrhythmia | none |
|  | Supraventricular tachyarrhythmias (level 4 SMQ) | Arrhythmia supraventricular  Atrial fibrillation  Atrial flutter  Atrial parasystole  Atrial tachycardia  Frederick’s syndrome  Sinus tachycardia  Supraventricular extrasystoles  Supraventricular tachyarrhythmia  Supraventricular tachycardia | ECG P wave inverted  Electrocardiogram P wave abnormal  Retrograde p-waves |
|  | Tachyarrhythmia terms, nonspecific (Level 4 SMQ) | Anomalous atrioventricular excitation  Cardiac fibrillation  Cardiac flutter  Extrasystoles  Tachyarrhythmia | none |
|  | Ventricular tachyarrhythmias (Level 4 SMQ) | Accelerated idioventricular rhythm  Arrhythmic storm  Cardiac fibrillation  Early repolarization syndrome  Parasystole  Rhythm idioventricular  Torsade de pointes  Ventricular arrhythmia  Ventricular extrasystole  Ventricular fibrillation  Ventricular flutter  Ventricular parasystole  Ventricular pre-excitation  Ventricular tachyarrhythmia  Ventricular tachycardia | none |
|  | Hypertension (Level 1 SMQ) | Accelerated hypertension  Blood pressure ambulatory increased  Blood pressure diastolic increased  Blood pressure inadequately controlled  Blood pressure increased  Blood pressure management  Blood pressure orthostatic increased  Blood pressure systolic increased  Catecholamine crisis  Diastolic hypertension  Essential hypertension  Hypertension  Hypertensive cardiomegaly  Hypertensive cardiomyopathy  Hypertensive crisis  Hypertensive emergency  Hypertensive end-organ damage  Hypertensive heart disease  Hypertensive nephropathy  Hypertensive urgency  Labile hypertension  Malignant hypertension  Malignant hypertensive heart disease  Mean arterial pressure increased  Neurogenic hypertension  Orthostatic hypertension  Prehypertension  Secondary hypertension  Supine hypertension  Systolic hypertension  Withdrawal hypertension | Aldosterone urine abnormal  Aldosterone urine increased  Angiotensin converting enzyme abnormal  Angiotensin converting enzyme increased  Angiotensin I abnormal  Angiotensin I increased  Angiotensin II abnormal  Angiotensin II increased  Angiotensin II receptor type 1 antibody positive  Blood aldosterone abnormal  Blood aldosterone increased  Blood catecholamines abnormal  Blood aldosterone increased  Blood catecholamines abnormal  Blood catecholamines increased  Blood pressure abnormal  Blood pressure ambulatory abnormal  Blood pressure diastolic abnormal  Blood pressure fluctuation  Blood pressure orthostatic abnormal  Blood pressure systolic abnormal  Catecholamines urine abnormal  Catecholamines urine increased  Ectopic aldosterone secretion  Ectopic renin secretion  Epinephrine abnormal  Epinephrine increased  Labile blood pressure  Metanephrine urine abnormal  Metanephrine urine increased  Non-dipping  Norepinephrine abnormal  Norepinephrine increased  Normetanephrine urine increased  pseudoaldosteronism  Renal vascular resistance increased  Renin abnormal  Renin increased  Renin-angiotensin system inhibition  Tyramine reaction |
|  | Myocardial infarction (Level 2 SMQ) | Acute cardiac event  Acute coronary syndrome  Acute myocardial infarction  Angina unstable  Blood creatine phosphokinase MB abnormal  Blood creatine phosphokinase MB increased  Coronary artery embolism  Coronary artery occlusion  Coronary artery reocclusion  Coronary artery thrombosis  Coronary bypass thrombosis  Coronary vascular graft occlusion  Kounis syndrome  Myocardial infarction  Myocardial necrosis  Myocardial reperfusion injury  Myocardial stunning  Papillary muscle infarction  Periprocedural myocardial infarction  Silent myocardial infarction  Troponin I increased  Troponin increased  Troponin T increased | Blood creatine phosphokinase abnormal  Blood creatine phosphokinase increased  Cardiac ventricular scarring  Coronary artery restenosis  ECG electrically inactive area  ECG signs of myocardial infarction  Electrocardiogram Q wave abnormal  Electrocardiogram ST segment abnormal  Electrocardiogram ST segment elevation  Electrocardiogram ST-T segment elevation  Electrocardiogram U wave inversion  Infarction  Myocardial necrosis marker increased  Scan myocardial perfusion abnormal  Vascular graft occlusion  Vascular stent occlusion  Vascular stent thrombosis |
|  | Other ischaemic heart disease (level 2 SMQ) | Acute cardiac event  Angina pectoris  Angina unstable  Anginal equivalent  Arteriosclerosis coronary artery  Arteriospasm coronary  Cardiac perfusion defect  Chronic coronary syndrome  Coronary angioplasty  Coronary arterial stent insertion  Coronary artery bypass  Coronary artery compression  Coronary artery disease  Coronary artery dissection  Coronary artery insufficiency  Coronary artery restenosis  Coronary artery stenosis  Coronary artery surgery  Coronary brachytherapy  Coronary bypass stenosis  Coronary endarterectomy  Coronary no-reflow phenomenon  Coronary ostial stenosis  Coronary revascularisation  Coronary steal syndrome  Coronary vascular graft stenosis  ECG signs of myocardial ischaemia  External counterpulsation  Haemorrhage coronary artery  Ischaemic cardiomyopathy  Microvascular coronary artery disease  Myocardial hypoperfusion  Myocardial hypoperfusion  Myocardial hypoxia  Myocardial ischaemia  Percutaneous coronary intervention  Prinzmetal angina  Stress cardiomyopathy  Subclavian coronary steal syndrome  Subendocardial ischaemia  Wellens' syndrome | Arterial revascularisation  Arteriogram coronary abnormal  Cardiac stress test abnormal  Cardiopulmonary exercise test abnormal  Cardiovascular event prophylaxis  Computerised tomogram coronary artery abnormal  Elastic vessel recoil complication  Electrocardiogram PR segment depression  Electrocardiogram ST segment depression  Electrocardiogram ST-T segment abnormal  Electrocardiogram ST-T segment depression  Electrocardiogram T wave abnormal  Electrocardiogram T wave inversion  Electrocardiogram U wave inversion  Exercise electrocardiogram abnormal  Exercise test abnormal  Post angioplasty restenosis  Restenosis  Stress echocardiogram abnormal  Vascular stent stenosis  Wall motion score index abnormal |

Abbreviations: AE-adverse event, SMQ-standard MedDRA query, PT-preferred term

Supplement Table 2. Most common adverse events in all of the MDMA reports in the FAERS database (FAERS Dashboard, accessed 3.20.2023).

| **Adverse Event/PT** | **Percentage** |
| --- | --- |
| Drug Abuse | 35.18% |
| Toxicity To Various Agents | 22.20% |
| Substance Abuse | 12.70% |
| Drug Dependence | 8.28% |
| Somnolence | 7.24% |
| Serotonin Syndrome | 7.15% |
| Drug Interaction | 6.96% |
| Intentional Product Misuse | 6.96% |
| Agitation | 6.87% |
| Overdose | 6.40% |
| Completed Suicide | 5.83% |
| Coma | 5.64% |
| Aggression | 5.55% |
| Poisoning | 5.08% |
| Mydriasis | 4.61% |
| Miosis | 4.61% |
| Loss Of Consciousness | 4.33% |
| Cardiac Arrest | 3.86% |
| Rhabdomyolysis | 3.76% |
| Pulmonary Oedema | 3.67% |
| Altered State Of Consciousness | 3.48% |
| Respiratory Arrest | 3.48% |
| Death | 3.39% |
| Drug Abuser | 3.39% |
| Alcohol Abuse | 3.20% |
| Vomiting | 3.10% |
| Tachycardia | 3.10% |
| Seizure | 3.10% |
| Cardio-Respiratory Arrest | 2.92% |
| Respiratory Depression | 2.82% |
| Suicide Attempt | 2.73% |
| Depressed Level Of Consciousness | 2.73% |
| Brain Oedema | 2.73% |
| Substance Dependence | 2.45% |
| Bradycardia | 2.35% |
| Euphoric Mood | 2.35% |
| Confusional State | 2.16% |
| Intentional Overdose | 2.16% |
| Road Traffic Accident | 2.16% |
| Off Label Use | 2.07% |
| Drug Withdrawal Syndrome | 1.79% |
| Suicidal Ideation | 1.69% |
| Withdrawal Syndrome | 1.69% |
| Depression | 1.60% |
| Headache | 1.51% |
| Abnormal Behaviour | 1.51% |
| Hypoglycaemia | 1.51% |
| Poisoning Deliberate | 1.51% |
| Acute Kidney Injury | 1.41% |
| Anxiety | 1.41% |
| Bradypnoea | 1.41% |
| Hypotension | 1.32% |
| Nervous System Disorder | 1.32% |
| Hyperthermia | 1.32% |
| Psychotic Disorder | 1.32% |
| Multiple Injuries | 1.32% |
| Renal Failure | 1.22% |
| Insomnia | 1.22% |
| Hypertension | 1.22% |
| Oxygen Saturation Decreased | 1.22% |
| Sudden Death | 1.22% |
| Hyperpyrexia | 1.22% |
| Nausea | 1.13% |
| Multiple Organ Dysfunction Syndrome | 1.13% |
| Hallucination | 1.13% |
| Disseminated Intravascular Coagulation | 1.13% |
| Asphyxia | 1.13% |
| Drug Diversion | 1.13% |
| Amnesia | 1.03% |
| Visual Impairment | 1.03% |
| Dehydration | 1.03% |
| Malaise | 1.03% |
| Hallucination, Visual | 1.03% |
| Intentional Product Use Issue | 1.03% |
| Pneumonia Aspiration | 1.03% |
| Victim Of Crime | 1.03% |
